# A Case Study on the Implementation of a Positive Youth Development Program (Project P.A.T.H.S.) in Hong Kong: Learning from the Experimental Implementation Phase

**DOI:** 10.1100/tsw.2008.125

**Published:** 2008-10-10

**Authors:** Tak Yan Lee

**Affiliations:** Department of Applied Social Studies, City University of Hong Kong, Hong Kong

**Keywords:** positive youth development, Project P.A.T.H.S., project implementation, process variables, continuous quality improvement, Chinese adolescents

## Abstract

This investigation of the implementation of a positive youth development program (Project P.A.T.H.S.) was part of a large study undertaken comprehensively to explore how effective the Tier 1 Program was in practice and how the results can shed light on future developments. Utilizing a case study approach, individual and focus group interviews were conducted in 2007 to examine the factors that influence the process and quality of implementation of the Tier 1 Program of the Project P.A.T.H.S. The focus of this study was on how the implementers of a school made use of the experience gained in the Experimental Implementation Phase (EIP) in 2005/06 to improve the program implementation quality in the Full Implementation Phase (FIP) in 2006/07. Results showed that the program implementation in the FIP was generally high and the program was well received by the implementers. Factors that facilitated the implementation of the program were identified, including the adoption of an incremental change strategy, the incorporation of the program into both formal and informal curricula, positive perceptions of the program among staff and agency social workers, sufficient school administrative support, excellent cooperation between the school and the social work agency, presence of a dedicated school contact person and instructors who engaged themselves in continuous quality improvement of the implementation, and an emphasis on application of what had been learned. Difficulties encountered by the school in the process of implementation were also observed. Based on the present findings, key process variables that facilitate or impede the implementation of positive youth development programs are discussed. Implications for future program implementation are also discussed.

### INTRODUCTION

The Project P.A.T.H.S. (**P**ositive **A**dolescent **T**raining through **H**olistic **S**ocial Programmes) is a holistic positive youth development program developed for junior secondary school students in Hong Kong[1,2]. The project has two tiers. While the Tier 1 Program is a universal program that utilizes a curricula-based approach for all Secondary 1 (S1) to Secondary 3 (S3) students of the participating schools, the Tier 2 Program is provided for students who have greater psychosocial needs. After 1 year of the Experimental Implementation Phase (EIP) in 2005/06, the Full Implementation Phase (FIP) was launched in 2006/07.

Although the effectiveness of the project has been documented in previous studies using different methods and from different sources, including objective outcome evaluation[3], subjective outcome evaluation[4,5,6,7], qualitative evaluation[8,9], process evaluation[10,11], and interim evaluation[12,13], there is a need to illuminate the process variables that influence the quality of program implementation. Despite the importance of quality of implementation in positive youth development programs, systematic research in this important area is far from adequate[14,15,16] in both international and Chinese contexts. Particularly lacking are studies that describe how well programs are implemented, as well as what factors inhibit or promote effective implementation[17,18,19].

The recent focus of school-based prevention programs has been to identify and replicate effective research-based programs, i.e., programs that have been tested rigorously and achieved positive results. Some programs have emerged as exemplary in meeting these criteria, and have been regarded as "works" that will then be put on the list designed for entities seeking to implement evidence-based programs[20,21,22,23,24]. Once an organization chooses a model program, it expects to achieve outcomes similar to those found in research trials, contingent upon being able to implement the program with integrity to the designed model[15].

Chen[25] provided a conceptual model for factors that influence implementation. These factors include characteristics of (1) the implementation system (i.e., process and structure of the implementation and training system), (2) the implementer (e.g., teacher and school staff), and (3) the setting in which the program is implemented (e.g., school climate, principal's support, and district support). In a comprehensive literature review, Shek and Sun[26] summarized five factors that influence the quality of program implementation, namely program, people, process, policy, and place (5 "P"s). These factors were examined in this case study.

The first element is a good **program**. The program should have the following attributes: well designed with clear goals; tailor made for adolescents' needs; socially and culturally sensitive; user friendly; with ready-made teaching materials; emphasis on practical competencies and generic skills; evidence-based program design; design conducive to instructors' willingness to implement the program as intended, without overlapping with other existing programs in school, and with novel and attractive activities[14,15,27,28,29].

Second, **people** are crucial to the effective implementation of school-based prevention programs[14,15,25,30]. Research studies showed that the attributes include the leadership of the school principal[31]; committed coordinators who offer ongoing support to the front-line teachers[15], foster communication among different parties, and provide clear guidance and information regarding the program implementation[14,20]; and training workshops for teachers to familiarize themselves with the program philosophy and content[15], the necessary teaching strategies, and the need for fidelity, as well as cultivate their enthusiasm and support for the program[14,32]. Research studies also showed that characteristics of the teachers, including rich teaching experiences, good preparation for teaching, possession of a graduate degree, and a greater sense of belonging to the school contributed to program effectiveness[29,33].

The coordinator is a key person that influences the quality of program implementation. Research findings showed that coordinators who were too far remote from the classroom (e.g., those in school district offices) were often not perceived as credible by teachers, and thus had difficulty communicating with instructors or offering assistance. On the other hand, enthusiastic local coordinators were able to provide the onsite monitoring and proactive problem-solving advice as compared to external research staff[15].

Teacher support for the program is crucial. While the majority of instructors have very positive views of the program, others may resent the mandate to teach. Research findings showed that teacher dissatisfaction sometimes resulted in instructors deviating from the curriculum by supplementing lessons (i.e., adding videos, "scare tactics", or other activities) or deleting information, activities, or entire lessons[15]. In order to support a program that utilizes valuable class time, teachers must believe that the program is worthwhile, have a sense of ownership for it, and feel supported by school administrators[34,35].

In terms of the school-based prevention program implementation **process**, seven attributes were found in research studies[14,15,29,30]. They are: (1) using interactive teaching strategies, (2) using facilitation skills to teach the program concepts and skills so as to generalize students' learning, (3) adherence to the designed program, (4) good classroom management skills, (5) good time management skills, (6) support of the principal in the implementation process, and (7) enthusiastic teachers who create a positive classroom atmosphere that raises the students' learning interest and motivation.

Moreover, effective delivery via effective teaching strategies is also instrumental to successful program implementation. Utilization of more interactive techniques, such as role play, demonstration, and behavior rehearsal, which were more conducive to implementation success than strictly lecturing, could reduce student boredom, problem behavior, and nonparticipation in class[14,15]. Furthermore, parental awareness and student behavior were related to the implementation quality[15].

Concerning school **policy**, elements of effective program implementation include integrating the program into the school formal curriculum, emphasizing program fidelity, existence of clear goals in the program implementation process, and good manpower deployment with division of labor to lessen the load of teaching and administrative duties[14,31]. Researchers also suggested that the school could arrange regular meetings with teachers to discuss problems encountered and the respective solutions, and to build up a supportive working environment as well as organize final evaluation meetings to applaud good work done, review past practices, and gain feedback[14].

Finally, a warm and supportive school environment ("**place**"), where the school principal, coordinators, and teachers have the same view for supporting the implementation of the program in the school, is conducive to effective program implementation. Chen[25] argued that although an intervention is the major change agent in a program, the "implementation system" is likely to make an important contribution to program outcomes. The implementation system provides the means and a context for the intervention, and is affected by a number of factors including characteristics of the implementers (i.e., "people" in this article), the nature of the implementing organization, and the quality of the linkages between this organization and the broader community[25]. Research findings supported that a nonauthoritarian working environment within the school that allows collaborative decision making and a positive atmosphere that enables job satisfaction will facilitate effective program implementation[33,36]. Furthermore, Eccles and Gootman[37] listed some physical features that promote positive youth development, including settings characterized with physical and psychological safety, appropriate structure, supportive relationships, opportunities to belong, positive social norms, support for efficacy, opportunities for skill building, and integration of family, school, and community efforts.

The strengths of case study research have been identified in the literature[31,38,39]. In this study, a case study was conducted in order to understand the contribution of the above factors to the quality of program implementation in the Chinese culture. There were two objectives of the case study: (1) to investigate the facilitating factors and obstacles encountered in the program implementation process from the program implementers' perspectives and (2) to draw implications for future program implementation.

Chen's[25] conceptual model for three key areas (i.e., the implementation system, the implementer, and the setting in which the program is implemented) was adopted to examine the case in light of the five categories of factors (i.e., program, people, process, policy, and place) that influence program outcomes.

## METHODS

### Participants

There were 52 schools and 207 schools that joined the Project P.A.T.H.S. (Secondary 1 Curriculum) in the EIP in the 2005/06 and FIP in the 2006/07 academic year, respectively. In the Tier 1 Program of the FIP, 112 of these schools adopted the full program (i.e., 20-h program involving 40 teaching units) and 95 adopted the core program (i.e., 10-h program involving 20 teaching units). Among these participating schools, six schools that joined the full program and one school that joined the core program were invited to participate in the case study. Based on the convenience sampling method, one school that had participated in both the EIP (taking the core program) and in the FIP (using the full program) was selected in this case study.

### Procedures

In this case study, a group interview with the school contact person and a focus group interview with one teacher and two agency social workers of the Tier 1 Program of the Secondary 1 Curriculum were conducted separately. Prior consent from the principal was sought for the school's participation. At the beginning of the interviews, informed consent was also obtained from the respondents, and the purpose of the study and the principle of confidentiality were repeatedly emphasized. The author, accompanied by a trained colleague who was studying a Master degree in Social Work with a Bachelor degree in Social Work, conducted the interviews. As the purpose of this case study was to examine the changes in school administrative arrangement in the two different phases and the implementation process of the Tier 1 Program (Secondary 1 Curriculum) in a retrospective manner, the interviews were conducted in early July 2007 after the school had completed the Tier 1 Program of the FIP. Specific self-constructed semi-structured interview guides for the interview and the focus group interview were designed to collect the data. There were two parts in each interview guide:

- Respondents' perceptions of the school administrative arrangement, including preparation, support within the school and from the social work agency, and program evaluation
- Respondents' perceptions of the program implementation process

In addition, the school was required to complete a school background information questionnaire with both closed- and open-ended questions in order to provide basic information about the school, the Tier 1 Program implemented in the school, instructors conducting the Tier 1 Program, and the implementation process of the FIP.

After data collection, the data were neatly typed and the recordings of the interviews were transcribed. Before data analysis, the raw transcription scripts were sent to the interview participants for their confirmation. No error was found in the precision of the dialogue in the transcribed texts. The procedures for data collection described above satisfied Principle 3 in the implementation of evaluation research proposed by Shek et al.[40].

### Data Analyses

The qualitative data were analyzed using general qualitative analyses techniques[41]. There were three steps in the process. First, words, phrases, and/or sentences that formed meaningful units at the raw response level were coded. Second, with reference to the questions of the interview guide, the codes were further combined to reflect higher-order attributes at the category of codes level. Third, the categories of codes were further analyzed to reveal the broader themes at the thematic level. Following the principles of qualitative analyses[40], the raw data and categorized data were kept in a systematic filing system for easy reference and audit.

### RESULTS

#### Summary of the School Characteristics

The interviewed school participated in the Tier 1 Program (Secondary 1 Curriculum) in the FIP in the academic year 2006/07. As shown in Table 1, there were 200 Secondary 1 students in five classes. The school adopted a double class teachers system. After the EIP, the school decided to adopt the 20-h full program (20 1-h sessions) instead of the core program (10 1-h sessions) and delivered the program through five different ways: class teachers' period, Life Education, weekly assembly, the Service Day, and project-based learning. In addition, a beginning session was organized for all Secondary 1 students in September 2006 and it was decided to conduct the conclusion session in September 2007 so that the conclusion session could be linked up with the ceremonial beginning session for the next academic year. The medium of instruction was Chinese. Although 12 teachers were involved in the actual delivery of the program, only half of them received training provided by the organizer. This was because the agency social workers assumed the tasks of preparing teaching materials, modifying the presentation materials provided by the organizer according to the needs of the students, delivering some of the program units for the first time in the presence of the teachers, and briefing the teachers on the contents and processes of the lessons before actual delivery. As it was very difficult for the teachers and social workers to meet together to prepare the lessons, this arrangement provided chances for those teachers who had not attended the training workshops to clarify the issues and discuss them with the social workers and other teachers. In contrast, the roles of the teachers included teaching the program, monitoring classroom discipline, coordinating the program-related matters, and following-up.

The school arranged different kinds of meetings in the program implementation process. Before implementation, there were five preparation meetings, where the implementation objectives and arrangements were discussed. With regard to ongoing cooperation between the social workers and the teachers, sharing meetings were held after school assemblies. The frequency was about once every 2 weeks. There were also one mid-term meeting and one final evaluation meeting. Issues discussed in the evaluation meetings included mode of cooperation, the division of labor, teaching materials, sharing about students' situations, and feedback.

The findings on the school administrative arrangements and related issues were divided into several parts.

a. **Support for the program and its rationales** — The school contact person revealed that the school offered Life Education for junior secondary students. She explained that the school principal highly commended the program because it was well designed to meet the needs of the students. According to the school contact person, the school principal strongly supported the project with a long-term view to integrate the program into the subject of Life Education in response to the education reform that was under way in Hong Kong[42,43,44]. The principal took the initiative to participate in the EIP, facilitated the teachers' involvement in as well as ownership of the program and support from the agency social workers, and supported the Tier 2 program through personal participation in some of the activities. The school contact person echoed that she considered the program to be especially helpful to junior secondary school students. According to the interviewees, strong support from the school contact person was evident as she played the roles of the planner, coordinator (S2 program), motivator, supporter, implementer, and evaluator of the program. Moreover, all interviewees considered that the program was good and its contents matched the needs of the students.

**TABLE 1**
**Summary of the Characteristics of the School**

| | |
|---|---|
| **I. Basic Information of the School** | |
| Total no. of S1 students (no. of S1 classes) | 200 (five classes) |
| Class teacher system | Double class teachers system |
| Participation in Life Education program organized by other organizations | No |
| No. of uniform/social and voluntary services groups in school | Three |
| Small groups, workshops, and seminars specifically arranged for S1 students | i. Leadership training; ii. Volunteer training; iii. Peer counseling scheme; iv. Environmental protection awareness; v. Mental health; vi. S1 orientation; vii. Promoting caring culture/antibullying behavior |
| **II. Basic Information of Tier 1 Program Participation** | |
| Hours | 20-h full program |
| Mode | Mode 3 (20 1-h sessions) |
| Program arrangement | Class teachers' period (30%, about once per week, totally 12 sessions); Life Education (50%, about once per week, totally 20 sessions); weekly assembly (10%, totally four sessions); Service Day (10%, totally four sessions); project-based learning |
| Rationales of the program arrangement | The Tier 1 program can be incorporated into the formal curriculum gradually with wider participation from teachers. |
| Medium of instruction | Chinese |
| Consolidation session | N.A. |
| Beginning session | Yes |
| Conclusion session | Yes (An award scheme and ceremony was held.) |
| Supplementary activity | Yes |
| Other activities | No |
| Student assessment of the P.A.T.H.S. program | Yes (Homework and class participation, no examination. Results will be indicated in students' academic reports.) |
| Have joined Experimental Implementation Phase | Yes (The experience gained from the EIP in the previous year helped the implementers to revise the details of the implementation in this year.) |
| **III. Information of the Instructors Conducting Tier 1 Program** | |
| No. of instructors who conducted Tier 1 Program | 12 Teachers (all of them volunteered to take part), two agency social workers |
| No. of instructors who joined P.A.T.H.S. training | Six teachers, two agency social workers |
| No. of instructors who joined the workshops on "Handling Behavioral and Emotional Problems of Students" | One teacher |
| No. of instructors who joined the workshops on "Personal Growth and Psychological Well-Being of Teaching Professionals" | Two agency social workers |
| **IV. Basic Information of Tier 1 Program Implementation** | |
| No. of preparation meetings and content | Five preparation meetings: briefing on program contents, distributing activity materials, briefing on how to run activities; two preprogram preparation meetings |
| No. of instructors who conducted Tier 1 Program in each class | Three instructors (two teachers and one agency social worker) |

*Table 1 continues*

**TABLE 1 (continued)**

| | |
|---|---|
| Person responsible for preparing the teaching materials and the way of preparation | The agency social worker modified the PowerPoint presentations and relevant materials according to the needs of the students. The worksheets were prepared and printed before the school term. The students were asked to keep all the worksheets in a folder. Additional worksheets were prepared by the agency social worker and delivered to teachers before class. |
| The format and frequency of lesson preparation among instructors/Reasons for not preparing the lessons together | It was very difficult for the teachers and social workers to meet together to prepare for lessons. In addition, teachers found that it was most efficient for the social workers to prepare and then brief them on the contents and processes of the lessons. Teachers had chances to clarify the issues and discuss with the social worker and other teachers. |
| The format and frequency of experiences sharing after class among instructors | The sharing meetings were held after school assemblies. The frequency was about once every 2 weeks. |
| No. of evaluation meetings and content | One mid-term evaluation meeting and one final evaluation meeting. Issues discussed in the evaluation meetings: mode of cooperation, the division of labor, teaching materials, sharing about students' situations, and feedback. |
| Cooperation between the school and social work agency | The social work agency was responsible for preparing for the P.A.T.H.S. proposal and financial management. Agency social workers ran some of the units at the first class and helped the other teachers grasp the key points and master the contents of the unit. |
| The role of teacher in Tier 1 Program implementation | i. Teaching the program; ii. Monitoring classroom discipline; iii. Coordinating the program-related matters; iv. Following-up |
| The role of social worker in Tier 1 Program implementation | i. Teaching the program; ii. Monitoring classroom discipline; iii. Preparing teaching materials; iv. Coordinating the program-related matters; v. Following-up. |

b. **Perceived program arrangement, including its advantages and disadvantages** — The school made changes in the program arrangement after gaining some valuable experience in the EIP. There were several significant changes in the program arrangement in the FIP.

- First, with regard to the program arrangement at the school level, there were three changes. The first one was the adoption of the full program in view of the experience gained from the EIP. As a result, the total number of sessions doubled from 20 to 40. The second one was the incorporation of half of the program into the curriculum of Life Education. The advantages were (i) the program contents met the needs of the students, (ii) the program topics could merge with the existing and the school-based curriculum, (iii) it reduced some teachers' workload in designing teaching materials for the assemblies, and (iv) it also streamlined the administration of the program implementation. On the other hand, there were disadvantages: (i) it demanded structural changes in the school administration to implement the program effectively; (ii) some previous tailor-made teaching materials prepared by the teachers could not be used; (iii) some subject teachers felt it difficult to be familiar with all their students due to time constraints; (iv) additional coordination work was needed; (v) it involved mainly three different parties to teach the program, i.e., class teachers, Life Education teachers, and agency social workers; and (vi) some program contents had to be revised to match students' special needs. The experience gained in the EIP provided sufficient groundwork for the school to plan for the implementation of the program in the next phase (FIP). The third one was that the school made use of the class teachers' periods to deliver 30% of the program. The advantages were (i) it involved junior secondary class teachers to deliver the program so that more teachers would be engaged, (ii) the program would be fully incorporated into the school curriculum in the long run, (iii) it created chances for the class teachers to be more familiar with all their students, (iv) the teachers used existing time slots for teaching, and (v) it did not increase the teachers' burden. However, the interviewees pointed out several disadvantages: (i) it required better coordination to oversee the implementation as more people were involved, (ii) it increased the stress of class teachers, and (iii) it created a big demand in training and manpower issues.

- The second significant change in the implementation was to integrate the beginning session for the incoming S1 students and the concluding session for existing S1 students into one event instead of two. Advantages of this change were (i) the two-in-one session created opportunities for reminding all and rewarding some of the S2 students, and setting peer role models for incoming S1 students at the beginning of the school year; and (ii) it saved manpower and reduced the workload of teachers. No disadvantages were observed.
- Third, the school kept using agency social workers to assume part of the teaching load in the Tier 1 Program. Having gone through the EIP, the advantages of such a practice were identified: (i) social workers were considered more appropriate to teach some of the topics; (ii) it alleviated the role conflict of teachers in teaching some sensitive issues, such as dating; and (iii) it reduced the workload of teachers to a certain degree. The disadvantages were (i) it increased the difficulty in coordination and (ii) social workers were not familiar with students.

The school contact person welcomed the changes in the program implementation as it was incorporated into the formal curriculum.

c. **Manpower deployment** — From the interviews, it was revealed that the school had mobilized different teaching staff to assist in the implementation. The manpower distribution in the Tier 1 Program was as follows: (i) one school contact person oversaw the program implementation and communicated with the Life Education teachers; (ii) two form (S1 and S2) coordinators supported teachers in doing preparation work, printing and distributing teaching materials, and acting as a bridge between teachers and social workers; (iii) 10 class teachers taught five S1 class teachers' periods; (iv) one specialist taught some topics in Life Education across S1 to S3; (v) one specific teacher taught project-based learning; and (vi) two social workers supported S1 and S2 class teachers by preparing for the teaching materials as well as teaching the first one or two classes for demonstration purposes. The school contact person revealed that the S1 coordinator had to assume other extracurricular activities, so coordination work in S1 experienced some difficulties.

d. **Facilities for implementation** — As revealed by the teacher and the social workers, the facilities in the school were sufficient and there was a user-friendly, computerized, room-booking system in school. A large special room was reserved to meet the need for space in order to run some of the activities. The teacher also revealed that a culture of students offering help to teachers was established. Teachers could easily find some students to help on some tasks. Moreover, the agency could provide further help in facilities as it was located near the school.

e. **Support within school and from the social work agency** — As revealed from the interviews, the school principal established a positive image of the Project P.A.T.H.S. among staff, students, and parents. He supported the staff to attend training workshops provided by the project team and encouraged ownership of the program among teachers. He was considered very supportive to the program. The school contact person was highly appreciated for her assumption of many roles for the planning, coordination, implementation, and evaluation of the program. Attributes of the school contact person included supportive with a clear sense of direction, cooperative, and encouraging collaborative decision making. According to the interviewees, qualities of the teachers were cooperative and willing to share experiences. However, a significant percentage of teachers did not receive the training prior to program implementation. The school contact person and the teachers perceived that the support from the social workers was sufficient and they were satisfied with it. It was evident that the school and its teachers had a good collaborative and trustful relationship with the agency social workers. Specially, the perceived support from social workers included (i) quick response to the needs of students, (ii) preparation of teaching and presentation materials based on the needs of schools, (iii) demonstration provided for teachers who were not familiar with the content, (iv) they were readily available at school due to physical proximity, (v) resourcefulness and trustworthiness, and (vi) other administrative support.

f. **Impression about the overall administration** — The school contact person was very satisfied with the manpower deployment in the EIP as the teachers volunteered to assume the role of program implementers. She was also satisfied with the communication among colleagues and the improvements made in administrative arrangement in the FIP. The teacher and the social workers also appreciated that the school contact person had followed through the program implementation diligently. The interviewees were satisfied with the overall program administration.
g. **Advice for administrative arrangements for the next academic year** — The school contact person, who also acted as the S2 curriculum coordinator, mentioned the need for training of the form coordinators. She also opined that it would be better if the subject teachers of Life Education were also class teachers.
h. **Other aspects** — Meetings within school: As revealed from the interviews, the implementers met on different occasions and for different purposes. Briefing meetings before the lessons were held by the social workers for the teachers. Other than the formal evaluation meetings, there were short evaluation meetings held among social workers and the teachers after teaching and periodic class management meetings among class teachers on students' behaviors and performance.

### Implementation Characteristics in the School a. **Teachers' and students' responses in the implementation process** — From the focus group interview, it was revealed that teachers in general had positive comments on the program. However, the students' involvement varied. Students liked activities and novel topics, and they felt bored when attending intensive sessions. Their learning motivation seemed to depend on the topics. Some students did not feel comfortable in sharing about themselves in class and some were not cooperative. Some were considered as having inappropriate learning attitudes because they knew that their performance in the P.A.T.H.S. program would not be assessed. Furthermore, some students considered that the program content was just common sense and challenged the teacher for time spent on the topics.
b. **Coteaching** — The focus group interview findings showed that the cooperation between the teachers and agency social workers was very good. They cooperated and trusted each other in coteaching. For example, social workers demonstrated how to run some activities, class teachers supported the social workers by sharing information about the students' background, social workers observed the students' performance in class and did follow-up work after the lesson, and social workers taught some programs that teachers did not feel comfortable enough to teach.
c. **Teaching techniques** — Several techniques were used by the teachers in the program implementation process: (i) encourage students to participate in the program through recognizing positive behaviors; (ii) motivate students according to their needs; (iii) encourage students to work on the worksheets through providing feedback; (iv) check students' assignments; (v) share personal experiences; (vi) draw students' attention through the use of carefully selected video vignette; and (vii) do not force students to share their feelings directly on some embarrassing issues and encourage them to make personal reflection during and after class.
d. **Handling students' worksheets** — The teacher felt that checking students' assignments was beneficial. Folders were also used to help students organize the worksheets.

e. **Student assessment in the Tier 1 Program** — The school contact person reported that Tier 1 Program assessment focused on application and was carried out in Life Education and project-based learning. Moreover, teachers gave feedback on the worksheets kept in the folder. No examination was needed for the class teachers' period.
f. **Difficulties encountered and solutions** — From the findings, the school contact person gave an account of how the school made use of the experiences gained in EIP. She was familiar with the program, had relevant teaching experience and sufficient training in the program, did good preparation, and provided tangible and emotional support to other teachers. With her dedicated work, minor difficulties encountered in the program implementation were straightened out. She did not perceive any big difficulties except that manpower planning and deployment were out of her control. She also pointed out that the lack of training of the S1 form coordinator, who had a heavy workload in extracurricular activities, led to coordination problems. With regard to the lack of training for some teachers, the agency social workers provided much help to protect the integrity of the program. The teacher revealed that some students challenged the necessity of teaching some "common sense" contents. The solution was for the teacher to discuss this with the class with an open mind and to help them understand the reasons for teaching them.
g. **Advice for implementation in the next year** — In the focus group interview, the social workers suggested that the project team should update teaching materials periodically and they would search for more relevant and latest information to meet the students' needs.

### Perceived Program Effectiveness, Program Success, and Overall Impression

Concerning the program effectiveness, the contact person did not think that she was in the appropriate position to judge the program effectiveness, although she could mention several cases of students who showed great improvement in their behavior. The teacher perceived that the program was beneficial to students, and it was a good opportunity for students to learn the subject matter and for teachers to get acquainted with the students. Generally speaking, interviewees were unsure about the actual changes in the students' behaviors. They thought that it was difficult to judge the effectiveness of the program at the moment, but they admitted that the program was helpful to students in that it provided an opportunity for students to reflect, and that the knowledge gained might be helpful in the future.

The informants perceived that the program was very comprehensive and well rounded in terms of program design and support, even though they provided no clear answer on program success. On the other hand, the interviewees quoted some examples of students keeping some teaching materials in their pockets for future reference. Social workers believed that the program represented a portfolio of students' personal growth and thus was beneficial to their growth. The contact person reflected that the program not only helped the students learn to develop positively, but also offered a good chance for the instructors to reflect on their own path of personal growth.

## DISCUSSION

### Factors that Facilitate or Impede Program Implementation

The purpose of the present paper was to identify major factors that influenced the quality and success of program implementation. Based on this case study, several factors were identified. The integrated interpretations presented above generate implications regarding future program implementation.

### Incorporation of the Program into Formal and Informal Curriculum: Incremental Changes

This case reveals that incorporation of the Tier 1 Program into the formal (Life Education) and informal (class teachers' period, assembly, Service Day) curriculum is feasible and practical. The school made slight changes in the existing manpower and administration structure, as well as the usual practice of the school, to make room for the incorporation of the Project P.A.T.H.S., a positive youth development program. Bella[45] pointed out that taking small steps toward change is a sensible way to improve the leadership and management practices in a school that must be able to make continuous improvements, without alienating staff by asking them to make drastic changes that seem overwhelming and unachievable.

Viewing the incorporation from the perspective of change management, the incremental change is ideal for several reasons. First, there is no need for a sudden change in the policy, administration, and manpower deployment. Second, the school can gradually involve more of its staff to engage in the delivery of the program content. Third, it takes time for the teachers to get ready in terms of receiving training and engaging in the program delivery. Fourth, the past decade of education reform in Hong Kong brought controversies and tensions between the education authority and the teachers, one of the major stakeholders. The pace of new initiatives introduced was criticized as being hasty. To remedy the situation, the government has taken some steps to reduce the workload and ease the pressure on teachers. With this background, the incremental change is considered appropriate. Finally, the school should adopt a progressive strategy in order to implement a new curriculum that requires changes in teachers' mind set and techniques.

### Positive Perceptions of the Program

As a prerequisite of successful implementation, the stakeholders must hold positive perceptions of the program. According to a study on the subjective outcome evaluation of the Project P.A.T.H.S., it was found that high proportions of the implementers had positive perceptions of the program and their own performance, and roughly 90% of the implementers regarded the program as helpful to the program participants[5].

The findings of this case study showed that all major stakeholders, including the principal, the school contact person who also served as a form coordinator, the teacher informant, and the two agency social workers, had positive perceptions towards the program goals and they regarded the program to be beneficial to student development. Moreover, voluntary participation in the program in the EIP tended to elicit less resistance and resentment. However, perhaps due to practical manpower deployment considerations, the school did not continue this practice in the FIP. This may be one of the reasons why the participation in the training course for the S1 program in the FIP was as low as 50%. With the tremendous support offered by the social workers, the school could manage to implement the program at a high quality. This laid a good foundation and exerted a significant influence on effective program implementation.

### Sufficient School Administrative Support

According to Mihalic et al.[15], it is necessary for principals to promote the program when adopting and integrating it into the school curriculum, as well as during implementation, in order to encourage enthusiasm from staff and ensure that the program has been delivered successfully. Moreover, active administrators must also introduce the program to teachers and elicit their support, attend training workshops, observe lessons, keep informed of implementation progress, and even teach classes in some cases[15].

In this case, the principal provided sufficient manpower for coteaching in both the EIP and the FIP consistently in order to enhance effective program delivery. Moreover, the teachers' voluntary participation in the program in the EIP, sufficient support from the social workers to teachers, and the use of existing structure to share the workload among teachers with specific roles and responsibilities for smooth program implementation were also the significant contributing factors. According to the interviewees, the principal engaged in discussion with teachers on the goals of the project with a view to integrating the program into school curriculum in the long run, as well as provided administrative support and involvement so as to engage the teachers to take part in the program before the EIP. Furthermore, although the principal was overwhelmed with administrative duties, he was willing to spend time visiting students while they were participating in outdoor activities organized by the social workers for the Project P.A.T.H.S.

### Excellent Cooperation between School and Social Work Agency

Bouck[46] demonstrated that conversations between coteaching partners are beneficial in order to address issues of roles, provide instruction, and handle classroom management and discipline, as well as issues such as loss of professional autonomy. Murawski and Dieker[47] pointed out that administrative support for coteaching at school is imperative and significant. In this case, the frequent and open communication between the social workers from the agency and teachers, the special roles and trust given to the social workers, and the availability of the social workers were considered remarkable. The school made good use of the resources provided by the Project P.A.T.H.S. to facilitate the collaboration and reduce some of the workload of the teachers. The collaborative efforts, as well as the complementary assistance in the program implementation, were contributing factors. The findings highlight the importance of deciding how to make use of the agency social workers to coteach the program and suggest future research on this aspect.

### Presence of a Dedicated School Contact Person

Reeves[48] reported the successful school management practices held by an award-winning school that emphasized teacher leadership and policies toward continuous improvement. The particular practice of intervention curriculum adjustments, teachers' efforts, and services in response to the program are considered as ideal examples of teacher leadership implementing positive change, and have led to remarkable progress for some of the most challenging students[48]. Gabriel[49] described those who build and maintain a successful team and facilitate professional growth of teachers as "teacher leaders". Lieberman[50,51] suggested that teacher leaders can build trust and rapport, manage resources, coordinate work, demonstrate expertise, and share knowledge with others. These findings provided support that the school contact person showed these qualities.

The school contact person strongly supported the project. She was very concerned with the implementation of the program in a serious manner, focusing on implementation progress and achieving solutions to implementation challenges. For example, she thoroughly studied all the curriculum units, took the initiative to assume the coordination work and streamline the administrative structure, and continuously provided emotional and tangible support to the teachers in formal and informal ways. Her insistence on continuous improvement was commendable. She displayed teacher leadership, which is a key to the school's strategies for continued success.

### Continuous Quality Improvement of the Implementation

There have been studies on identifying and diffusing effective research-based prevention programs. However, there is still much to be learned about the common problems faced when implementing the program in "real" settings, outside the rigorous controls of research trials[14,15]. This school made use of the experience gained in the EIP to plan for the changes in the administrative structure to facilitate the implementation. In the FIP, the school contact person was responsible for the liaison with S2 class teachers of the EIP and formed a group under the auspices of a school management committee that took care of discipline, guidance, moral and civic education, as well as career guidance issues. The frequent briefing and open sharing meetings among the instructors reflected the presence of a culture for continuous quality improvement.

### Emphasis on Application

From the interviews, the informants emphasized the importance of the application of what students learned from the Project P.A.T.H.S. Although there was no written examination for the students, the students were expected to learn how to apply, but not memorize, the concepts. The encouragement of reflection was also emphasized. This played an important part in the teaching-learning process. This emphasis was vital to improve the students' learning and classroom participation.

### Addressing the Obstacles and Barriers Encountered during Implementation

Mihalic et al.[15] pointed out that most sites for implementing school-based programs faced common barriers, including finding room in the school schedule for the program, gaining full support from key participants (i.e., site coordinators, principals, and program instructors), ensuring teacher participation in training workshops, and classroom management difficulties[15]. Their study also suggested some important factors that organizations should consider to ensure fidelity, such as selecting programs with features that minimize complexity while maximizing flexibility. Time constraints in the classroom should also be considered when choosing a program. Student behavior also influences program delivery, so schools should train teachers in the use of classroom management skills. The present findings concur with their views. According to the informants, Project P.A.T.H.S. involved comprehensive program monitoring and technical assistance that facilitated the identification and resolution of problems, and contributed to the overall high quality of implementation. Schools should recognize the importance of training and technical assistance to ensure quality program delivery.

### Implications of the Findings for Future Implementation of the Program

As far as the implications of the present findings for future implementation of positive youth development programs are concerned, several conclusions can be highlighted. First, the adoption of an incremental change strategy is important to the successful implementation of a school-based program. Second, the incorporation of the program into both formal and informal curricula, instead of one single subject, has advantages. However, further research is needed in order to examine the conditions for success. Third, the constructive effects of positive perceptions of the program held by the program implementers were illustrated in the present case study. Fourth, sufficient school administrative support is vital. Fifth, the exceptional collaboration between the school and the social work agency is commendable. Also, their hard work and their passion about adolescents are indispensable for successful program implementation. Sixth, the presence of a dedicated school contact person is another necessary condition for successful implementation. Seventh, striving for continuous quality improvement of the instructors and the school contact person should be essential. Finally, an emphasis on application of what has been learned should be the hallmark of all positive youth development programs.

### Limitations of the Case Study

Despite its rich content, there are some limitations of the present study. First, as a convenience sample, generalization of the findings is not desirable. However, this case study documented the school's successful experience of the program implementation to provide food for thought for other schools. Second, as the present interviews only involved four informants, the data collected would be more comprehensive if interviews with the school principal, teachers with special responsibilities to care for students' personal growth and special needs, form teachers, and the school social worker were involved. Due to the busy schedule and heavy workload, the intended interviewees could not attend the focus group. Third, the case study was conducted in a retrospective manner, and it would be more valuable if periodic interviews with the instructors and observations of the program delivery in classes were conducted as well. However, that would then cause further disruption of the normal operation of the school. Fourth, the presence of interviewers may have affected the responses of the respondents (e.g., social desirability effect) in the face-to-face interviews. However, as the transcribed texts were sent to the interviewees for review and the informants were encouraged to voice their views (including those that were negative in nature), this method of data collection was justified. Finally, in the data analyses, coding and categorization were done by a trained research assistant and cross-checked by the author. Although this method was justified for a small-scale case study, inter- and intrareliability tests were not conducted. Despite the above limitations, the present findings are generally coherent with the evaluation findings generated from different sources and different methods[3,4,5,6,7,8,9,10,11,12,13].

### ACKNOWLEDGMENTS

The preparation for this paper and Project P.A.T.H.S. were financially supported by The Hong Kong Jockey Club Charities Trust.
